# Diabetic Control Outcomes Amongst Patients Who Had Bariatric Surgery in a District General Hospital

**DOI:** 10.7759/cureus.5651

**Published:** 2019-09-13

**Authors:** Joshua Fultang, Ugochukwu Chinaka, Abdulmajid Ali

**Affiliations:** 1 General Surgery, University Hospital Ayr/University of West of Scotland, Ayr, GBR; 2 General Surgery, University Hospital Ayr/University of West of Scotland, Ayr, GBR

**Keywords:** type 2 diabetes mellitus, bariatric surgery, gastric bypass, laparoscopic sleeve gastrectomy, diabetes- diabetes in remission- complete remission- weight loss

## Abstract

Bariatric surgery remains the most effective weight loss treatment. It leads to significant and sustained weight loss and improvement in various metabolic diseases such as type 2 diabetes (T2DM). This piece of work aimed to investigate the remission of T2DM amongst patients who had laparoscopic Roux-en-Y gastric bypass (RYGB) and sleeve gastrectomies (LSG). This was achieved by carrying out a retrospective review of prospective data of 82 T2DM diabetic patients who had above bariatric surgery at the University Hospital Ayr from 2010 to 2016. Outcomes were assessed at two years postoperatively and documented. The main outcome measure was based on the achievement of partial or complete remission.

The average age of patients in this study was 49.6±8.1 with 52% female (n=49) and 48% male (=33). Preoperative body mass index (BMI) averaged at 42.6±6.2 kgm^2^. The majority (n= 43) of cases had a Roux-en-Y gastric bypass (RYGB) while (n=39) had laparoscopic sleeve gastrectomy (LSG). The average glycated haemoglobin (HbA1c) was 6.7±1.8 units. Fourteen patients who had diet-controlled diabetes were excluded. Of the patients left (n=68), partial or complete remission was achieved by 73.3% (n =50). Remission rates following RYGB, 87.2% (n=43) were higher than those following LSG (55.2%). Age, duration of diabetes, and HbA1c showed a statistically significant difference amongst both cohorts. No statistically significant difference was seen in BMI both at referral and at surgery between both cohorts of patients.

We concluded that preoperative BMI plays a very limited role in determining which patients go into remission in the short-term postoperative phase.

## Introduction

Diabetes mellitus (DM) is a metabolic disorder in which there is an aberration in the secretion and/or action of insulin [1]. This leads to abnormal carbohydrate metabolism characterized by hyperglycemia. Chronic hyperglycemia is associated with significant microvascular and macrovascular complications [2]. These complications have a great impact on the patient’s health and quality of life, and their management poses a significant financial strain on the National Health Service (NHS) [3].

Approximately 415 million people have DM and 193 million have undiagnosed DM worldwide [4]. In the UK, the number of people diagnosed with diabetes has more than doubled in the last two decades. It is now estimated that 6.6% of the population is affected with DM. The number of people diagnosed with DM is now recorded to be 3.7 million, 1.9 million more diagnoses as compared to 1988. A further one-million people are thought to have undiagnosed DM [5]. It is predicted that more than 5million will be living with DM by 2025 in the UK [6].

The major modifiable risk factor of developing mainly DM type II is being overweight or obese. Obesity accounts for 80%-85% of the risk of developing type II DM (T2DM) [7].

With rising trends in the prevalence of diabetes and obesity locally and worldwide, there is an ongoing debate about the most effective way to manage the cohort of patients who are diabetic and obese. Management of chronic hyperglycemia is achieved by a combination of lifestyle and medical and surgical strategies (bariatric surgery) [8]. However, bariatric surgery has been irrefutably shown to lead to superior rates of remission and cure of DM when compared to medical and lifestyle strategies in the short, mid, and long term [9-11].

Laparoscopic sleeve gastrectomy (LSG) and Roux-en-Y gastric bypass (RYGB) are currently the most performed metabolic surgery procedures in diabetic patients worldwide [12]. Laparoscopic adjustable gastric band (LAGB) and biliopancreatic diversion with duodenal switch (BPD+DS) are both carried out less frequently. The most carried out procedures under the National Health Service (NHS) are LAGB, RYGB, and LSG [13].

This piece of work aims to investigate and document the remission of T2DM amongst patients who had laparoscopic gastric bypass and sleeve gastrectomy in a district general hospital. The outcome measures focused on the relevance of age, duration of diabetes, HBA1c, and BMI in predicting remission will be explored.

## Materials and methods

This involved a retrospective analysis of data from 82 T2DM patients who underwent either laparoscopic sleeve gastrectomy (LSG) or Roux-en-Y Gastric Bypass (RYGB) between 2010 and 2016. All surgeries were carried out at the University Hospital Ayr, Scotland. Data were collected by reviewing the online Microsoft Access (Microsoft Corporation, Redmond, Washington) bariatric database and Scottish Care Information (SCI) Diabetes platform (SCI-DC, Scotland) for all patients who underwent bariatric surgery in Ayrshire and Arran. Outcomes were assessed two years post-surgery.

The patients were grouped into two cohorts based on achieving partial/complete remission or not. The modified American Diabetic Association's criteria define remission as achieved HbA1c levels of <5.7% without the ongoing use of pharmacological or surgical therapy for one year. Partial remission was defined as HbA1c levels of 5.7% to 6.5%, again in the absence of any ongoing therapy.

Age, duration, diabetes, glycated hemoglobin (HBA1c) at referral, BMI at surgery, and referral were analyzed in both cohorts. A t-test was used to determine the significance of the data.

Exclusion criteria

Patients with diet-controlled T2DM were excluded.

## Results

**Figure 1:**
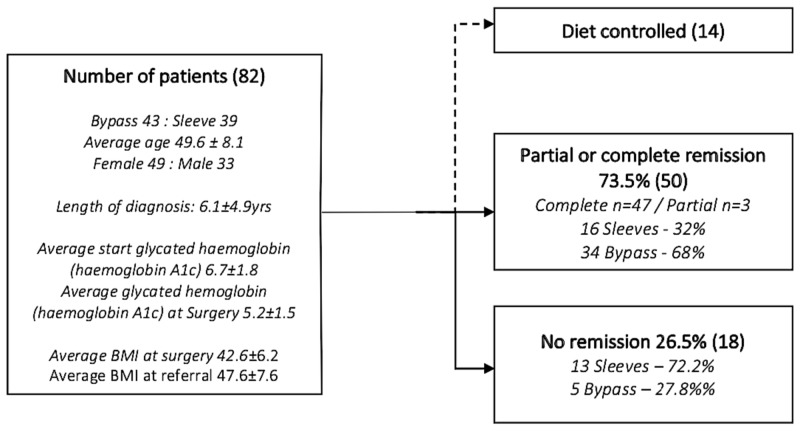
Showing summary of data from patients involved in the study Seventy-three percent of patients achieved partial or complete remission. Three patients with partial remission and 47 with complete remission.

Data from 82 T2DM patients who underwent bariatric surgery between 2010 and 2016 were identified and reviewed. Age, average BMI, and HBA1c are detailed in Figure 1. Of these patients, 14 had diet-controlled diabetes (Figure 1). Forty-three (43) of the 68 cases underwent RYGB and 39 had LSG (Table 1). The average age of patients in this study was 49.6±8.1 years with 52% (n=49) being female and 49% (n=33) male. The average BMI at surgery was 42.6±6.2 kgm^2^. A majority (58%) had RYGB while 42% had LSG. The average HbA1c at referral to the bariatric services was 6.7±1.8. Partial or complete remission was achieved by 73% (n=50) within two years postoperatively. Ninety-four percent of the 50 patients (n=47) and 6% (n=3) had complete and partial remission respectively. Two out of the three patients who achieved partial remission within two years had an RYGB and the other LSG, respectively.

**Table 1:** Showing type 2 diabetes mellitus remission rates following Roux-en-Y Gastric Bypass and laparoscopic sleeve gastrectomy at two years post-surgery Remission rates were higher at 2 years post-Roux-en-Y Gastric Bypass compared to laparoscopic sleeve gastrectomy

Procedure	Remission	No remission
Roux-en-Y Gastric Bypass (RYGB) n = 43	87.2%	12.8%
Laparoscopic Sleeve Gastrectomy n=39	55.2%	44.8%

Sixty-eight percent of the patients who went into remission had an RYGB and 32% an LSG. A total of 18 patients amongst whom 72.2% had LSG (n=13) and 27.8% RYGB (n=5) showed no significant improvement still requiring antidiabetic medications two years post-surgery. Of these, two went into remission at six months and restarted pharmacological therapy at 18 months postoperatively.

**Figure 2:**
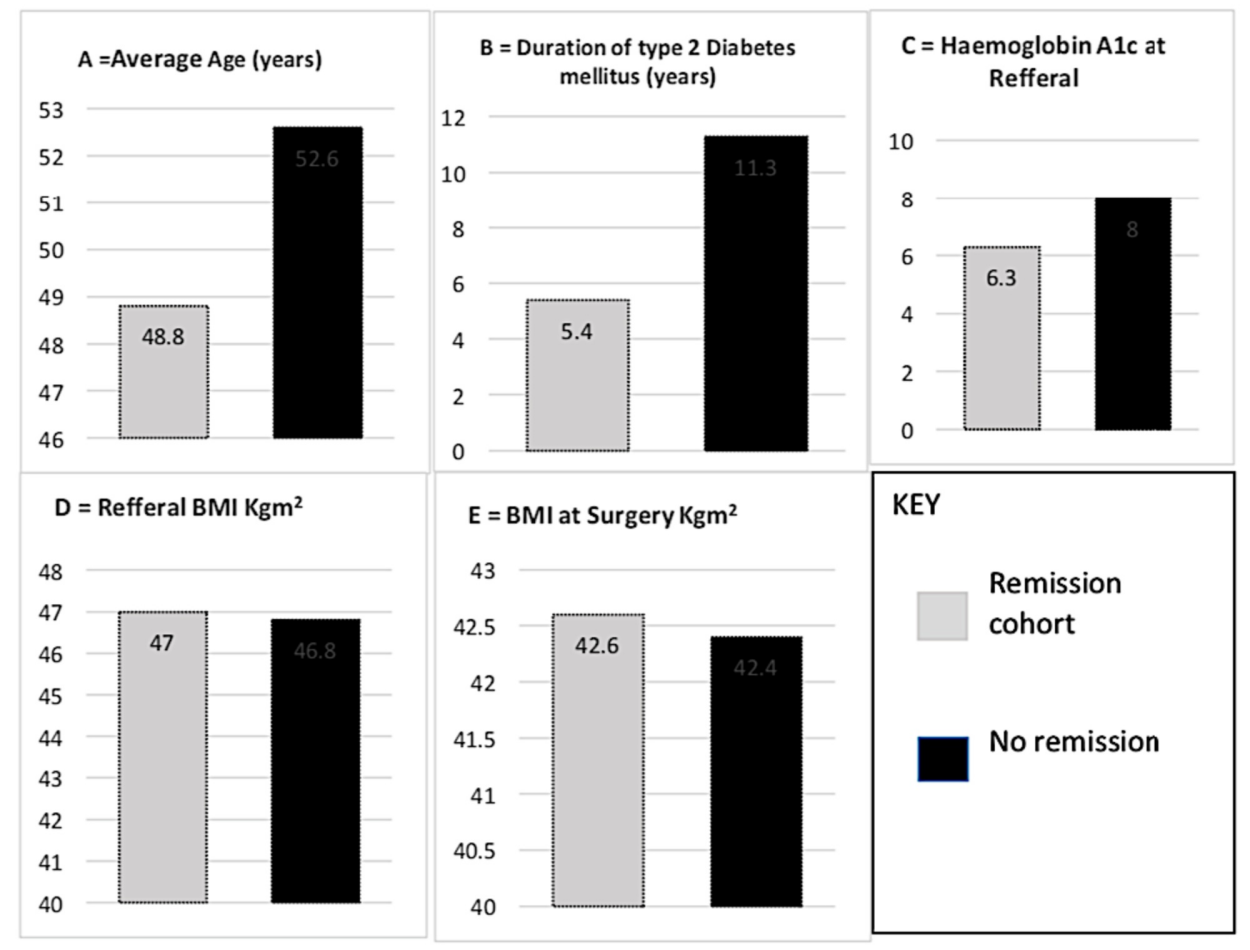
Showing the difference in both cohorts based on achievement of remission A = Average age = Remission 48.8±6.6 years / No remission 52.6±4.4 years – p-value 0.03 B = Duration of type 2 diabetes millitus = Remission 5.4±4.5 years / No remission 11.3±3.5 – p-value <0.01 C = Average hemoglobin A1c = Remission 6.3±2.2 / No remission 8.0±0.5 – p-value <0.01 D = BMI at Referral = Remission 47.0±7.6 / No remission 46.8 ±8.7 – p-value 0.92 E = BMI at surgery = Remission 42.6±6.6 / No remission 42.4±6.3 – p-value 0.91

Comparing both cohorts in terms of age, duration of diabetes, and BMI, as seen in Figure 2, the average age of those who did not go into remission was 52.64±4.4 years, which was higher than 48.8±6.6 of those who went into remission. There was a statistically significant difference in age between both groups (p-value 0.03). A similar observation was noted when looking at the duration of diabetes. As seen in Figure 2B, the duration of diabetes was 5.4±4.5 years and 11.3±3.5 years in the remission and no remission cohort. The HBA1c in the cohort of patients who went into remission was significantly lower (6.3±2.2 units) than those in the cohort who did not go into remission (8.0±0.5) units.

All patients with diet-controlled diabetes had normal HBA1c at less than six months postoperatively and this was maintained after two years. Four of these patients had an RYGB and 10 an LSG.

## Discussion

Bariatric surgery remains the most effective weight loss treatment. It leads to significant and sustained weight loss and amelioration of the patient’s physiological status. This, in turn, results in cardiovascular, metabolic [14], renal, and psychological benefits [15].

Our results show that complete or partial remission was achieved in up to 73% of diabetic patients who underwent bariatric surgery. This is comparable to the remission rate of 68.8% seen by Pucci et al. in 2018 [16] for both LSG and RYGB at two years postoperatively.

The adapted American Diabetic Associations definition of remission put forward by Ana M Ramous-Levi et al. in 2013, where remission was achieved if HbA1c levels were <5.7% without the ongoing use of pharmacological or surgical therapy for one year. Partial remission was defined as HbA1c levels of 5.7% to 6.5%, again in the absence of any ongoing therapy [17].

Most of the patients who achieved remission had an RYGB (68%), whilst 72.2% of those who did not achieve remission had LSG. This agrees with published data in which RYGB (restrictive and malabsorptive surgery) has higher short-term T2DM remission.

The remission rates of 87.2% seen following RYGB in the short term is similar to that found by Chikinuwo et al. in 2010 (89.9%). Short-term remission rates of 55% following an LSG are also comparable with published data [18]. Milone et al. (2013) also found that 66.7% of patients who underwent LSG went into remission at one year and 87.5% of patients who underwent restrictive and malabsorptive surgery [19].

Furthermore, we observed that those who did not go into remission were older patients and had a longer duration of diabetes (52.64±4.4 years and 11.3±3.5 years, respectively). Some have argued that both parameters are similar, as older patients turn to have a longer length of diagnosis [18].

There was also a statistically significant difference in the HbA1c at referral between both cohorts (6.3±2.2 units and 8.0±0.5 units). This may suggest that patients who went into remission probably had relatively better glycemic control preoperatively as compared to those who did not.

Interestingly, BMI was found to play a very limited role in determining those who went into remission in the short term. There was no statistical significance in BMI both at referral to the bariatric service and BMI at surgery, as seen in Figure 2D-2E. This raises the question of the use of BMI as a pivotal eligibility criterion for the cohort of patients who are obese and diabetic.

## Conclusions

Bariatric surgery is an effective therapy for T2DM, with better remission seen with RYGB as compared to LSG. Preoperative BMI does not have any impact on diabetes remission while older age and longer duration of diabetes have less improved outcomes. We recommend that T2DM patients receive bariatric surgery early for better outcomes.
